# Genome-Wide Microsatellite Identification in the Fungus *Anisogramma anomala* Using Illumina Sequencing and Genome Assembly

**DOI:** 10.1371/journal.pone.0082408

**Published:** 2013-11-27

**Authors:** Guohong Cai, Clayton W. Leadbetter, Megan F. Muehlbauer, Thomas J. Molnar, Bradley I. Hillman

**Affiliations:** Department of Plant Biology and Pathology, School of Environmental and Biological Sciences, Rutgers The State University of New Jersey, New Brunswick, New Jersey, United States of America; University of Ottawa, Canada

## Abstract

High-throughput sequencing has been dramatically accelerating the discovery of microsatellite markers (also known as Simple Sequence Repeats). Both 454 and Illumina reads have been used directly in microsatellite discovery and primer design (the “Seq-to-SSR” approach). However, constraints of this approach include: 1) many microsatellite-containing reads do not have sufficient flanking sequences to allow primer design, and 2) difficulties in removing microsatellite loci residing in longer, repetitive regions. In the current study, we applied the novel “Seq-Assembly-SSR” approach to overcome these constraints in *Anisogramma anomala*. In our approach, Illumina reads were first assembled into a draft genome, and the latter was then used in microsatellite discovery. *A. anomala* is an obligate biotrophic ascomycete that causes eastern filbert blight disease of commercial European hazelnut. Little is known about its population structure or diversity. Approximately 26 M 146 bp Illumina reads were generated from a paired-end library of a fungal strain from Oregon. The reads were assembled into a draft genome of 333 Mb (excluding gaps), with contig N_50_ of 10,384 bp and scaffold N_50_ of 32,987 bp. A bioinformatics pipeline identified 46,677 microsatellite motifs at 44,247 loci, including 2,430 compound loci. Primers were successfully designed for 42,923 loci (97%). After removing 2,886 loci close to assembly gaps and 676 loci in repetitive regions, a genome-wide microsatellite database of 39,361 loci was generated for the fungus. In experimental screening of 236 loci using four geographically representative strains, 228 (96.6%) were successfully amplified and 214 (90.7%) produced single PCR products. Twenty-three (9.7%) were found to be perfect polymorphic loci. A small-scale population study using 11 polymorphic loci revealed considerable gene diversity. Clustering analysis grouped isolates of this fungus into two clades in accordance with their geographic origins. Thus, the “Seq-Assembly-SSR” approach has proven to be a successful one for microsatellite discovery.

## Introduction

 Microsatellites (also known as simple sequence repeats, SSR), are stretches of DNA consisting of tandemly repeated short units, usually 1-6 base pairs. They are valuable tools in many research areas, such as population biology, genome mapping, and the study of genealogy, because they are multi-allelic, inherited co-dominantly, usually abundant, and cover most or all parts of the genome. The traditional method to develop microsatellite markers, which is still used by many labs today, generally involves the following steps: enrich microsatellite-containing sequences from sheared genomic DNA; clone the microsatellite-enriched DNA; extract plasmids; sequence the inserts through Sanger sequencing; design primers; and screen individual loci. The whole process can require several months of work and considerable resources. In fungi, which is the subject organism of this study, the traditional approach is even more time- and resource-consuming, because fungal species usually have lower densities of microsatellite loci and the alleles are often shorter with fewer polymorphisms, compared to many other organisms [1]. 

 Advances in sequencing technology are changing many aspects of the biological sciences, including methods to develop microsatellite markers. The high-throughput and low cost of next-generation sequencing enables the efficient generation of large amounts of genome sequence data from which microsatellite markers can be identified. The 454 sequencing platform (454 Life Sciences, Roche) has been used most frequently for this purpose to date, due to its production of longer reads of DNA. Using the 454 platform, many microsatellite-containing reads have sufficiently long flanking sequences to allow the design of primers to amplify the target microsatellite loci [2-7].

 In contrast, the Illumina sequencing platform generates shorter reads, but recent progress extends read length up to 250 bp on the Illumina MiSeq platform and up to 150 bp on other platforms (www.illumina.com). Furthermore, the read length can be extended with paired-end sequencing. Overall, the Illumina platform operates with much higher throughput and lower cost than the 454 platform; thus, it has become an attractive sequencing platform for use to identify large numbers of microsatellite loci. Castoe et al. [8] used paired-end sequencing to generate 114 bp x2 reads for a python and 116 bp x2 reads for two bird genomes. They searched for microsatellites on these reads and designed primers using their flanking sequences. This “SEQ-to-SSR” approach proved to be valuable, but with two caveats. The first caveat was that many reads did not have long enough flanking sequences to allow primer design (e.g. microsatellite loci were located toward one end). For example, in the three species they examined, primers were successfully designed for only 32.7% to 40.1% of the loci. In comparison, a 454-generated library allowed successful primer design for 49.6% of the loci. The second caveat was the need to identify and filter out primers that could amplify multiple PCR products. The authors alleviated this challenge bioinformatically by counting the occurrence of the primers in the dataset. 

 In this study, we used an alternative approach to Castoe et al. (2012). Instead of trying to design primers directly from the Illumina reads, we first performed a genome assembly of the host organism to achieve longer contiguous sequences, and then performed the microsatellite search and primer design based on the genome assembly, essentially a “SEQ-Assembly-SSR” approach. While genome assembly requires access to more sequence data than that needed in the approach used by Castoe et al. [8], advances in Illumina technology, in terms of higher throughput and lower cost, make this approach readily applicable. For example, at current prices, a 250 bp x2 paired-end sequencing run on the MiSeq platform costs approximately $1000 for reagents and produces 7.5-8.5 Gb of sequences, which is sufficient to provide several times coverage for most large genomes. Further, multiplexing (genomic libraries from different samples have different barcodes) allows multiple small genomes to be sequenced in a single run. The Illumina Genome Analyzer IIX and HiSeq platforms have even lower per-base sequencing cost than MiSeq (www.illumina.com). Our approach can be applied either for the development of microsatellite markers alone or in combination with genome sequencing projects that produce much deeper genome coverage.

 We applied our approach to the fungus *Anisogramma anomala* (Peck) E. Müller (Ascomycota: *Diaporthales*), which is the causal agent of the hazelnut (*Corylus* spp.) disease eastern filbert blight (EFB). In the United States, the European hazelnut, *C. avellana* L., is produced commercially, mostly in the Willamette Valley of Oregon. In the late nineteenth and early twentieth centuries, efforts to establish commercial production of European hazelnut in the Northeast region failed due to EFB [9-13]. The pathogen is endemic on American hazelnut, *C. americana* Marsh., an understory shrub commonly found in deciduous forests across a wide region of eastern North America [14]. While *A. anomala* causes insignificant cankers on the American hazelnut, it is lethal to the commercially important European species. On the latter, cankers expand at rates of up to 1 m per year [15], girdle branches, and result in canopy dieback and tree death in 5-12 years if control methods are not taken [16].

 To combat this disease threat, the state of Oregon established a quarantine in 1922 prohibiting importation of *Corylus* plants from east of the Rocky Mountains [13]. Nevertheless, this disease appeared in an orchard of European hazelnut in southwest Washington in the late 1960s [17] and reached Oregon’s Willamette Valley in 1986 [18]. The pollinizer cultivar ‘Gasaway’ was found to carry a dominant allele at a single locus that confers complete resistance to the Oregon strain of *A. anomala* [19]. In recent years, a number of cultivars carrying the ‘Gasaway’ resistance locus have been released [20-24]. Scientists and growers worry that the single-gene resistance may not be durable, and certain isolates of the fungus from east of the Rocky Mountains have indeed been shown to cause cankers on cultivars expressing ‘Gasaway’ resistance [25,26].

 Little is currently known about the population biology and genetic diversity of *A. anomala*, with only very limited molecular data available for this pathogen. Such information is vital to support disease resistance breeding efforts in hazelnut, which are ongoing in multiple locations in the United States [27]. In a search of GenBank conducted in October 2013, only four sequences for *A. anomala* and two sequences for *A*. *virgultorum*, the other species in the genus, were found, and all were ribosomal RNA gene sequences. An important reason for the lack of molecular data is likely that *A. anomala* is strictly obligate and biotrophic and thus very difficult to work with in the laboratory. This fungus is known to reproduce only in living host tissue through the production of sexual ascospores. Asexual spores have not been observed. Preliminary genome analysis identified candidate sequences for both MAT1-1 and MAT1-2 idiomorphs of mating type genes (unpublished), suggesting that this fungus is likely homothallic. It was suggested that *A. anomala* produces self-inhibitors, preventing ascospore germination and hyphae growth on artificial media. Only media amended with bovine serum albumin or activated charcoal were shown to support prolonged growth and only when inoculated with ascospores excised from infected plant tissue [28]. Even on these media, the growth is extremely slow. It takes several months to obtain only limited amounts of fungal material for DNA extraction, and no one has yet reported successful subculture. Here we report the development of a genome-wide database of microsatellite markers in *A. anomala* using Illumina sequencing and the “SEQ-Assembly-SSR” approach. 

##  Results and Discussion

### Genome sequencing and assembly

An Illumina paired-end library with a mean insert size of 420 bp was made using an isolate of *A. anomala* collected from Oregon State University (OSU), Corvallis, OR. The *A. anomala* library shared a single lane on a flow-cell with another library and was sequenced on the Genome Analyzer IIX platform to produce 151 bp x2 paired-end reads. A total of 26,036,313 reads, including 12,468,172 x2 in pairs (95.8%) were generated for the *A. anomala* library (SRA: SRX341929). The reads were shown to be of high quality, with even the last base having a mean quality score of over 33 (not shown). Excluding the 5 bp custom barcode located at the beginning of each read, there were 146 bp usable sequences.

 The *A. anomala* genome was assembled using SOAPdenovo [29] with K-mer size 51. The final assembly parameters are listed in Table 1. We achieved a contig N_50_ of 10,384 bp and a scaffold N_50_ of 32,987 bp. The assembled genome was surprisingly large, calculated to be almost 337 Mb if gaps in the scaffold were counted and over 333 Mb if gaps were excluded. 

**Table 1:** Summary of *Anisogramma anomala* genome assembly using SOAPdenovo [29].

	GC ratio	N_50_	N_max_	Total size
Contigs before gap-filling	34%	4,101	31,925	340,480,068
Scaffolds (>= 200 bp)	32%	32,987	223,344	336,895,534
Contigs after gap-filing	32%	10,384	74,811	333,579,400

To our knowledge, this represents the largest fungal genome sequenced to date [30]. Flow cytometry experiments using the same isolate provided a genome size estimate of approximately 370 Mb, supporting the assembly results (unpublished data). Based on the flow cytometry data, the Illumina sequences provided 10x coverage and 90% of the genome was represented in the assembly. We are currently generating more sequences and the full genome characterization will be presented in a separate paper. Here we focus on the development of microsatellite markers and their potential use for the study of genetic diversity and population structure of *A. anomala*.

### Construction of a genome-wide microsatellite marker database for *Anisogramma anomala*

**Figure 1:**
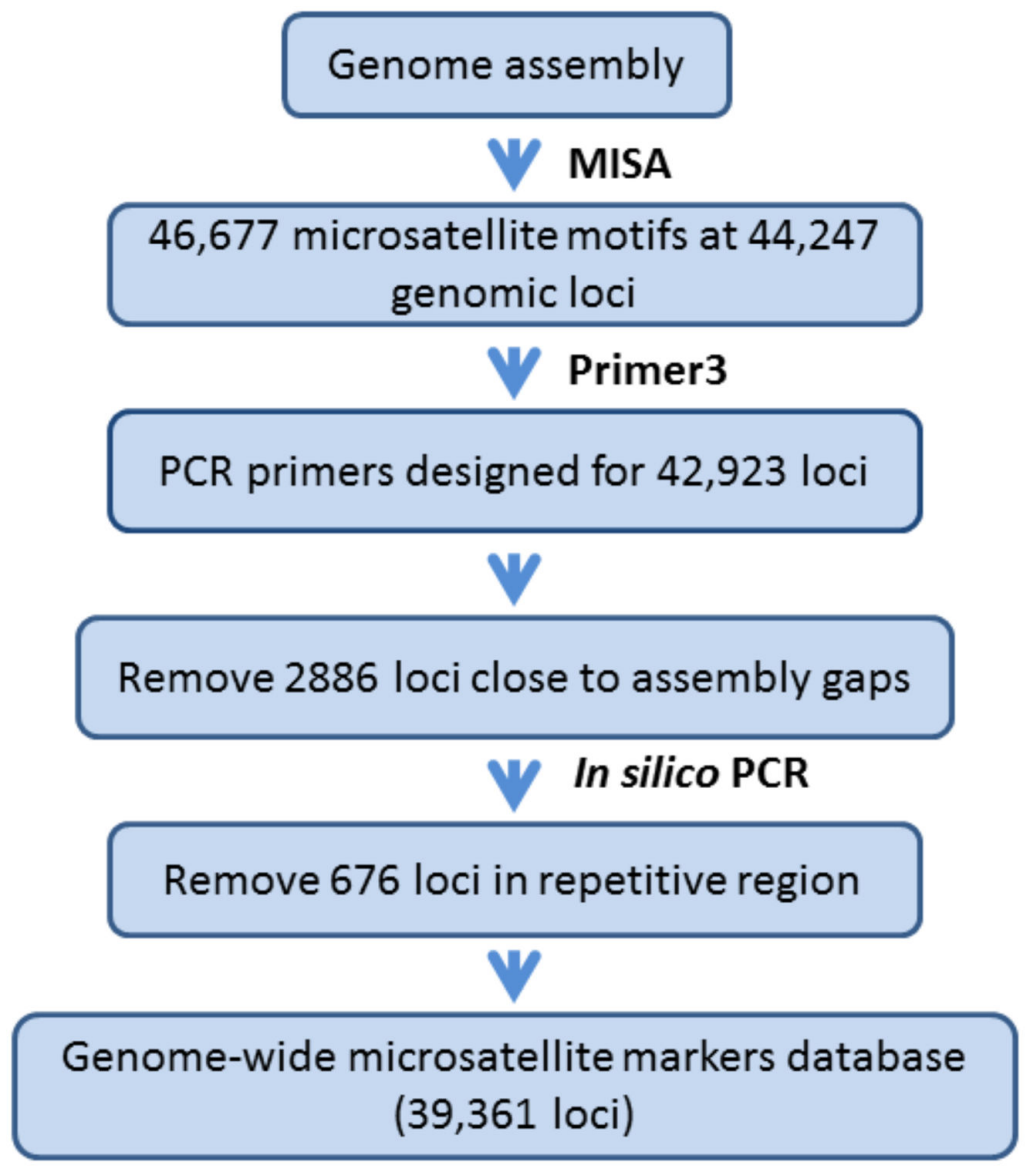
Flow chart describing the bioinformatics pipeline used to construct a genome-wide microsatellite marker database for *Anisogramma* *anomala*. See text for detail.

 The flow chart presented in Figure 1 illustrates the steps used to construct the genome-wide database of microsatellite markers in *A. anomala*. The genome assembly was fed to the PERL script MISA [31] to search for microsatellite motifs (unit size 1-6 bp). For a unit size of 1 bp, the minimal repeat number required was 10; for a unit size of 2 bp, the minimal repeat number required was 6; and for a unit size of 3-6 bp, the minimal repeat number required was 5. A total of 46,677 motifs were identified at 44,247 loci, including 2,430 compound loci in which two motifs were located within 100 bp from each other. Overall, this constitutes 141 loci per Mb genome. 

 Primer3 [32] was used to design primers from sequences flanking each locus. Primer pairs were successfully designed for 42,923 out of the 44,247 loci (97%). This result is much higher than the 32.7% to 40.1% achieved with un-assembled Illumina reads and the 49.6% achieved with un-assembled 454 reads [2]. We subsequently chose the best primer pair produced by Primer3 for each locus. 

 Some microsatellite loci were very close to the end of a contig. As a result, there were not enough flanking sequences on one side of the loci to allow proper primer design. For these loci, one of the primers was designed based on the sequence of the neighboring contig in the same scaffold. Because the sizes of the gaps in the assembly were estimated, the expected amplicons may be longer or shorter than estimated. Thus, these loci were considered less reliable than other loci for which both primers were designed based on the sequence of the same contig. Consequently, a total of 2,886 such loci were removed from the database. It should be noted, however, that most of these loci are likely valid and could be explored if needed. 

 Certain microsatellite loci may be part of longer repetitive sequences, and as such, primers used to amplify these loci may amplify multiple targets. We ran In-Silico PCR to detect these loci. Based on this analysis, a total of 676 primer pairs were predicted to produce two or more amplicons and were removed from the database.

 The final database includes 39,361 loci. A complete database, including an ID, type and exact sequence of individual microsatellite motifs, sequences and Tm of forward and reverse primers, and the size of expected amplicon in the sequenced Oregon strain, is included in Table S1. 

### Characterization of microsatellite motifs in *Anisogramma anomala*

**Figure 2:**
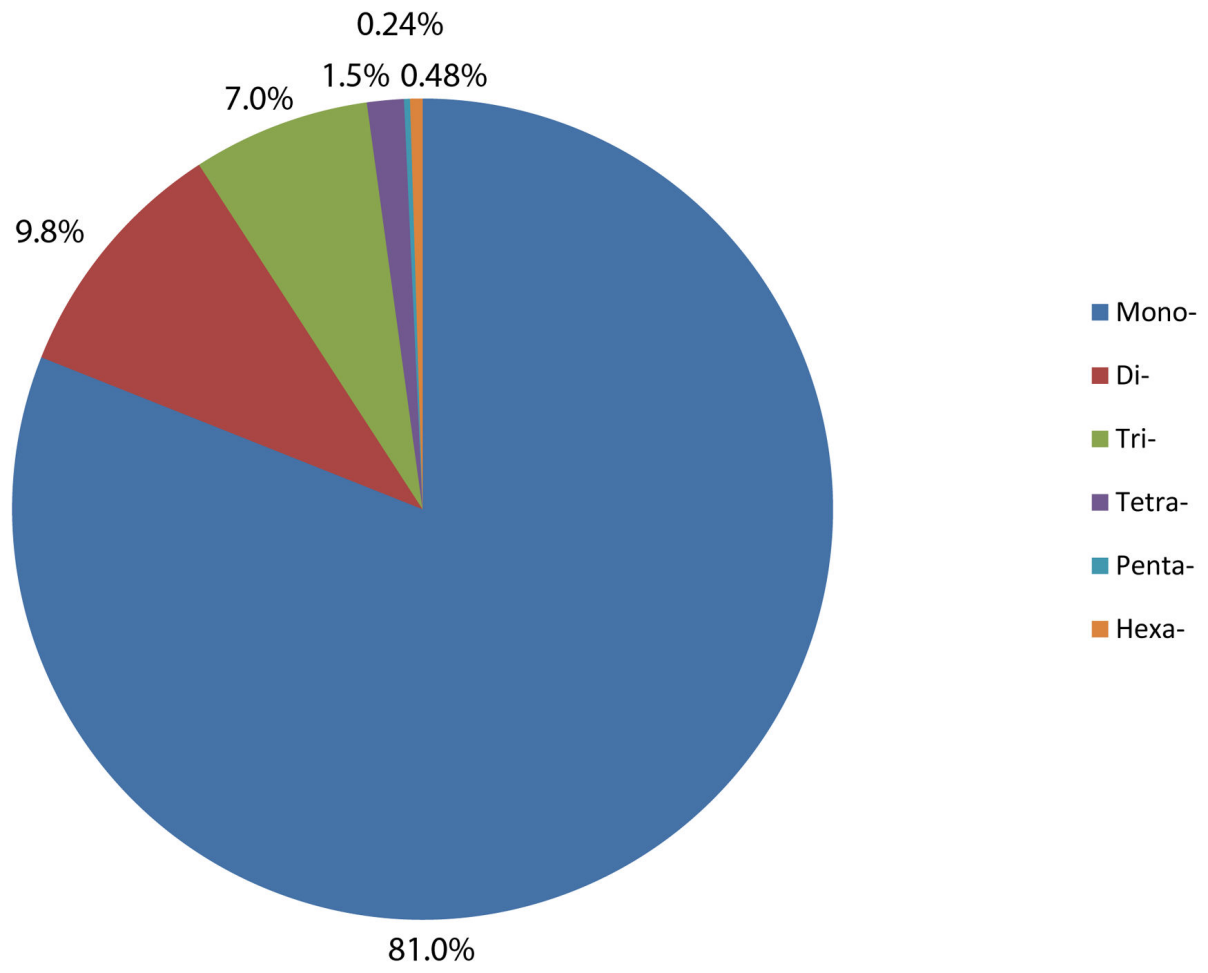
Frequency distribution of microsatellite loci in *Anisogramma anomala* by motif length.

The identified microsatellite motifs in *A. anomala* consist of 37,824 mono- (81.0%), 4,575 di- (9.8%), 3,264 tri- (7.0%), 677 tetra- (1.5%), 111 penta- (0.24%), and 226 hexa- (0.48%) nucleotide motifs (Figure 2). The number of tri-nucleotide motifs is 71% of that of the di-nucleotide motifs, and the number of hexa-nucleotide motifs is more than twice that of the penta-nucleotide motifs. The over-representation of tri- and hexa- motifs may be explained by contribution of coding sequences—these two types of motifs are usually over-represented in coding regions likely because they do not interrupt the open reading frames [33]. 

**Figure 3:**
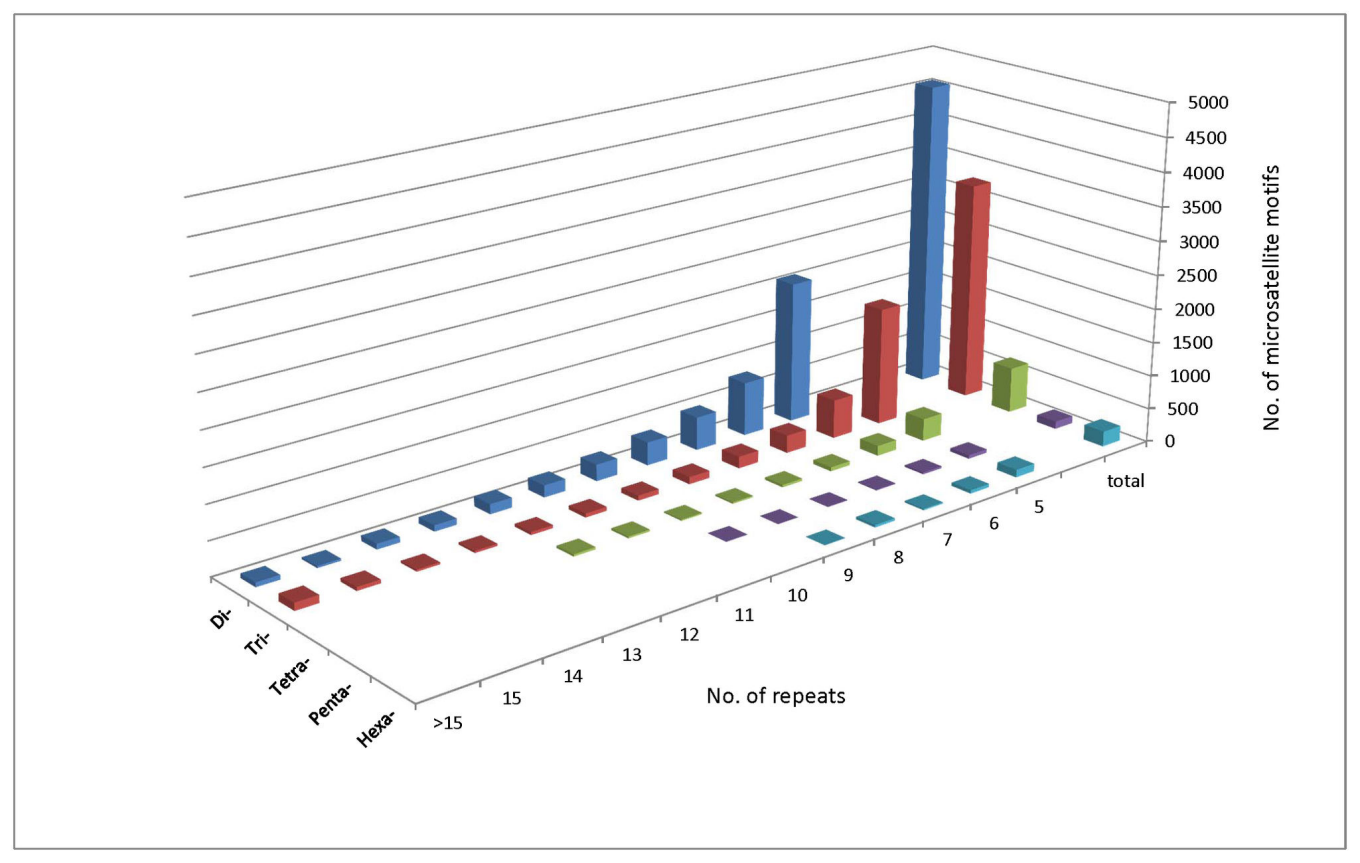
Distribution of Di- to Hexa- microsatellite motifs in *Anisogramma* *anomala* by number of repeats.

 The distribution of microsatellite motifs in *A. anomala* with regard to the number of repeats was examined (Figure 3 and Table S2). As expected, the number of motifs decreased as the repeat number increased. Up to 73 repeat units for mono-nucleotide motifs, 24 for di-nucleotide motifs, 17 for tri-nucleotide motifs, and 12 or fewer for longer motifs were observed. 

**Figure 4:**
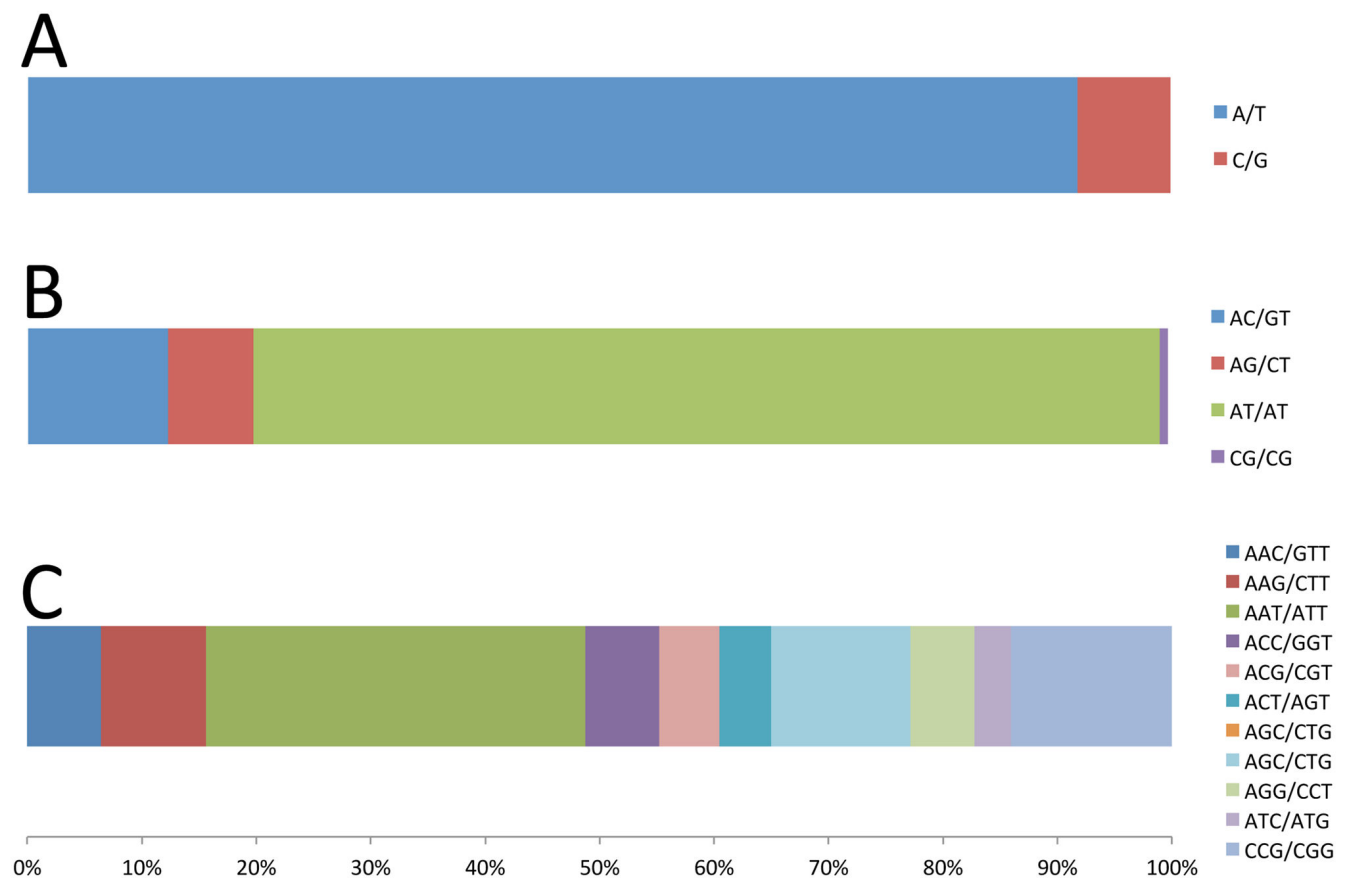
Frequency distribution of mono-, di-, and tri- microsatellite motifs in *Anisogramma* *anomala* by motif sequences.

 To analyze individual motif types, shifted motifs and their reverse-complement motifs (e.g. ATC/GAT, TCA/TGA, and CAT/ATG) were grouped together. Results showed the *A. anomala* genome has high A:T ratio (68%). If microsatellites have no sequence preference, we would expect AT-rich motifs to be more common. However, this was not always the case. In mono- to di- motifs, AT-rich motifs dominate; whereas in tri- motifs, the AAT/ATT motif is most common (1,081, 23.6%), the CGG/CCG motif is second (458, 10%), being more common than any other tri- motifs (Figure 4). A similar situation is observed in longer motifs—while the AAAT/ATTT motif dominates the tetra-motifs, the motifs ACAGC/CTGTG and ACCAGC/CTGGTG, both GC-rich, are most common in penta- and hexa- motifs, respectively (Table S2). 

### Experimental screening and preliminary population study

**Figure 5:**
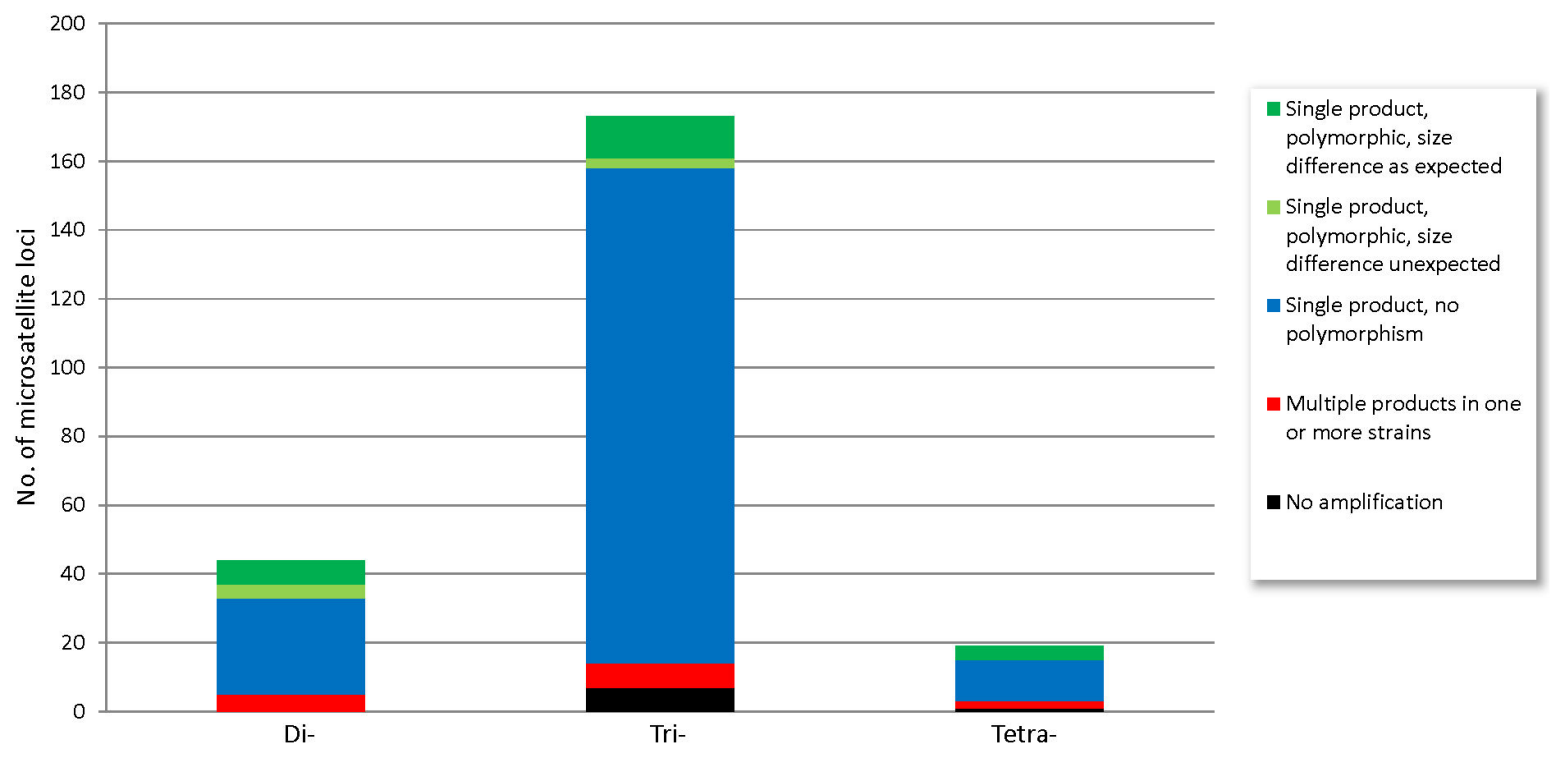
Experimental screening of microsatellite loci in *Anisogramma* *anomala*. For polymorphic loci, the expected size difference between alleles is multiples of the unit size (e.g. 2x, 3x, and 4x for di-, tri- and tetra- nucleotide motifs, respectively), assuming the motifs are perfect and there are no size differences in the flanking regions.

To evaluate the quality of our database and to identify polymorphic, informative loci, we performed first-pass screening of 236 loci with di- (44), tri- (173) and tetra- (19) nucleotide motifs (Table S3). Four geographically representative *A. anomala* isolates, from New Jersey, Ohio, Oregon, and Wisconsin, respectively, were used to detect potential polymorphisms. The Oregon strain used in the screening was isolated from a different sample than the strain used in genome sequencing. PCR amplifications were successful for 228 (96.6%) of the loci (Figure 5), suggesting that most primer pairs in the database will amplify their targets. 

 The results showed that single products were produced for 214 of 236 (90.7%) loci (Figure 5). Most of these loci (184) did not show polymorphisms among the four isolates tested. For the polymorphic loci, it is expected that the size differences among the alleles are representative of multiples of the unit size (2x, 3x, and 4x for di-, tri- and tetra- nucleotide motifs, respectively), if the motifs are perfect and there are no size differences in the flanking regions. Among the 30 polymorphic loci identified, 23 (9.7% of the total loci) showed these expected size differences. For the other seven loci, an assumption was made that either there are size differences in the flanking regions or the motifs in some of the strains are not perfect, or both.

 Multiple products were observed in one or more isolates for 14 (5.9%) loci (Figure 5). Based on the genome size estimate from flow cytometry, the genome assembly represents approximately 90% of the genome. Repetitive sequences present challenges for genome assembly, especially when using only short reads generated using next-generation sequencing technologies. As such, the missing 10% of the genome is likely rich in repetitive sequences. It is likely that In-Silico PCR did not eliminate all of the primer pairs that amplify multiple targets because highly similar copies of the target sequences are present in the genome but not necessarily in the genome assembly. 

**Figure 6:**
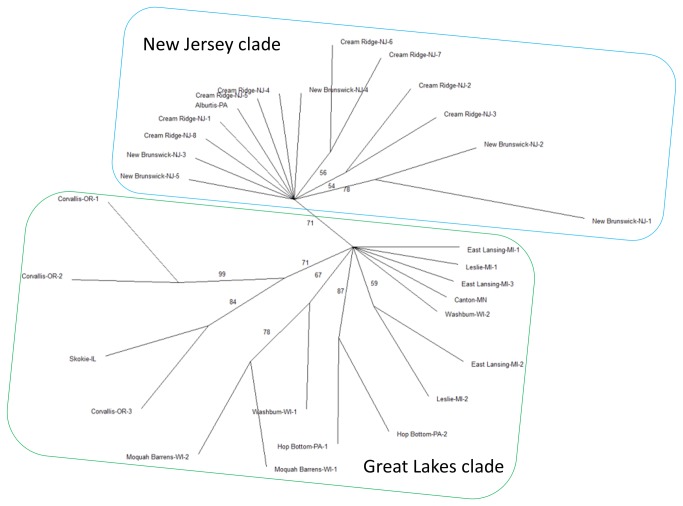
Consensus UPGMA tree of 30 *Anisogramma* *anomala* isolates characterized by 10 microsatellite loci. Isolates are named by the city and state from where they were collected, and followed by a number if multiple isolates were obtained from the same location. Isolates were grouped into two clades in accordance with their geographic origins. UPGMA trees were generated by PowerMarker version 3.25 [36] using frequency-based genetic distance of shared alleles [39]. Consensus tree was computed from 100 bootstrap replicates using “consense” program in software package PHYLIP version 3.695 (http://evolution.genetics.washington.edu/phylip.html). Numbers by the branches are bootstrap support values in 100 replicates. Branches with less than 50% bootstrap support were collapsed.

We are in the process of screening more loci and using the polymorphic loci to study the population structure of *A. anomala* in North America based on hundreds of isolates. In a preliminary population study, 30 isolates were examined using 11 perfect polymorphic loci identified above. Statistics of individual loci are summarized in Table 2. At a 1 percentile *P*-value threshold, only one pair of markers, Aa00689 and Aa00940, were found to be in linkage disequilibrium, and the former was removed from further consideration. Clustering analysis grouped the isolates into two clades: one includes isolates from New Jersey (the New Jersey clade), the other includes isolates from Illinois, Michigan, Minnesota and Wisconsin, all of which are states surrounding the Great Lakes (the Great Lakes clade) (Figure 6). An important question is whether these two clades represent natural populations of this fungus. Pennsylvania may represent the interface of these two clades. An isolate from Alburtis, PA, approximately 30 miles from the nearest New Jersey border, clustered with the New Jersey clade; whereas, approximately 100 miles to the north, two isolates from Hop Bottom, PA clustered with the Great Lakes clade. Isolates from Oregon, believed to be descendants from an introduction from east of Rocky Mountains, clustered with the Great Lakes clade, specifically, an isolate from Skokie, IL. However, since *A. anomala* has a long, symptomless latent period [16] and hazelnuts have been disseminated widely around the U.S. and Canada as ornamental and backyard garden plants (for example, the popular and very EFB-susceptible contorted hazelnut *C. avellana* ‘Harry Lauder’s walking stick’), one cannot exclude the effects the movement of infected plant material may have had on the population structure of the fungus. This small-scale population study served as a proof-of-concept showing that the “Seq-Assembly-SSR” approach was successfully applied to the *A. anomala*, and the informative markers that were identified can be readily used in population studies. It also provided a first snapshot of the population structure of this fungus. We are awaiting data from many more isolates, characterized by more loci, to conduct a thorough analysis and provide fine mapping of *A. anomala* population structure from which we can draw much stronger conclusions. 

**Table 2:** Summary statistics of 11 polymorphic microsatellite loci in 30 isolates of *Anisogramma anomala*.

Marker	Motif	Major allele frequency	Sample size	# of alleles	Gene diversity**^a^**	Polymorphism information content**^b^**
Aa00170	(GTT)_n_	0.6071	30	3	0.5026	0.4072
Aa00689	(TCA)_n_	0.5417	30	4	0.5868	0.5146
Aa00940	(TTA)_n_	0.6667	30	2	0.4444	0.3457
Aa01096	(TTTA)_n_	0.8519	30	2	0.2524	0.2205
Aa01290	(TAA)_n_	0.6667	30	2	0.4444	0.3457
Aa01430	(TAC)_n_	0.8462	30	2	0.2604	0.2265
Aa02342	(TTC)_n_	0.4000	30	4	0.6733	0.6111
Aa02555	(TTA)_n_	0.8077	30	2	0.3107	0.2624
Aa02768	(AAT)_n_	0.5333	30	2	0.4978	0.3739
Aa03169	(ATA)_n_	0.9259	30	2	0.1372	0.1278
Aa16574	(CT)_n_	0.4138	30	4	0.6540	0.5852

^**a**^ Gene diversity, also referred to as expected heterozygosity, is the probability that two randomly chosen alleles from the population are different. It was calculated according to Weir [37].

^**b**^ Polymorphism information content is a modification of gene diversity, from which it subtracts the additional probability that an individual does not contribute information in a linkage analysis. It was calculated according to Botstein, et al. [38].

### Concluding remarks

Next-generation sequencing technologies have revolutionized the development of microsatellite markers. The 454 platform, with its longer reads, understandably became the first choice in earlier studies. The high-throughput and lower per-base cost of the Illumina platform now argue for its favor, especially when used to generate large numbers of genome-wide microsatellite loci. Its shortcoming, in comparison to the 454 platform, is its shorter reads. However, technologies now exist to circumvent this limitation. Here, we successfully applied to the fungus *A. anomala* the novel “SEQ-Assembly-SSR” approach, which first required genome assembly from the raw Illumina sequence reads, then identification of microsatellites from the draft assembly. This approach overcame the read length obstacle and resulted in primer pairs successfully designed for 97% of the identified loci, with 96.6% of the designed primer pairs amplifying their targets. In contrast, in a previous study using unassembled sequence reads, primers were designed for only up to 40.1% loci. While differences in primer requirements and other aspects may account for some of the differences between the studies, the much longer contig lengths achieved by genome assembly (N_50_ 10,384 bp) is likely the main reason. Furthermore, the “SEQ-Assembly-SSR” approach allows the use of *in vitro* PCR to remove many primer pairs that will amplify multiple targets from the genome. 

The experimental screening of individual loci remained expensive and time consuming. For the 236 loci we screened, primers alone (including FAM-labeled M13 primer) cost $3,904 (Integrated DNA Technologies). Running the samples on the sequencer in our internal core facility and other reagents/consumables cost over $3,000. If more loci were screened and if replicates were included, the cost and time input would proportionally increase, soon becoming prohibitive. In future studies, we plan to use direct sequencing to identify polymorphic microsatellite loci. For example, a 250 bp x2 paired-end sequencing run on the MiSeq machine will provide 7.5-8.5 Gb data, which is enough to provide 7x coverage for three *A. anomala* isolates. The reads can then be mapped to the assembled draft genome to identify polymorphic microsatellite loci. Reagent cost for library making, sequencing, and other consumables will be less than $1,500, and the experiment can be carried out within one week. In this approach, all of the microsatellite loci present in the genome can be screened, and the bioinformatic pipeline can be used for other organisms. 

## Materials and Methods

### Culture and DNA extraction

 Infected hazelnut branches containing mature stromata of *A. anomala* were kindly provided by Dr. Shawn Mehlenbacher. The branches were harvested in winter 2009 from plants growing at the OSU Smith Horticultural Research Farm, Corvallis, OR, that were inoculated 18 months prior in the greenhouse with a local isolate of *A. anomala*. The branches were cut into 5 cm sections and surface-sterilized by vigorously shaking in 10% bleach (0.525% sodium hypochlorite) for 3 min and in 70% ethanol for 30 sec, followed by a rinse in sterile H_2_O after each treatment. The stromata were then hydrated in sterile H_2_O for 30 min. After surface-drying under a laminar flow hood, the tops of the stromata were cut off with a sterile razor blade to expose the necks of the perithecia. At this point, a white viscous matrix containing asci and freed ascospores was visible. Another sterile razor blade was inserted under the stromata and was used to provide pressure from below to push the white matrix out of perithecial neck. This matrix was then picked up by a sterile fine needle. 

 The matrix that contained asci and ascospores was collected in sterile H_2_O containing 10 ppm rifampicin and 100 ppm streptomycin. The suspension was vortexed for 1 min to release individual ascospores. The ascospore concentration was determined with a hemocytometer and adjusted to 1x10^5^ per ml with sterile H_2_O containing the same antibiotics. Half a milliliter of the spore suspension was used to inoculate solid medium overlaid with sterile cellophane in standard Petri dishes. One liter of medium contained 2.7 g modified Murashige and Skoog basal salt mixture [34], 20 g sucrose, 2 g yeast extract, 2 g L-Asparagine, 15 g Bacto agar, 0.25 g activated charcoal, and 10 mg Rifampicin (added after autoclaving)[28].

 The cultures were grown at 18 °C in the dark for 2 months, by which time many spores had germinated and grown into opaque, whitish colonies ~0.25-0.5 mm in diameter. Mycelium was harvested by rinsing the cellophane in sterile H_2_O. DNA was extracted using a DNeasy plant kit (Qiagen) according to manufacturer’s instructions. 

### Genome sequencing and assembly

An Illumina paired-end library was made according to manufacturer’s instructions except a 5-bp customer barcode was added. The mean insert was 420 bp excluding adapter sequences. The library was sequenced sharing a single lane with another library on the Genome Analyzer IIX platform to produce 151 bp x2 paired-end reads. 

 Sequencing quality and nucleotide distribution were explored using the fastx toolkit (http://hannonlab.cshl.edu/fastx_toolkit/) installed on a local Linux server. The 5-bp barcode was trimmed and reads with “N”s were filtered out. The genome was assembled with SOAPdenovo [29] using the trimmed and filtered reads. After thoroughly testing K-mer size, the value 51 was chosen because it gave the best contig N_50_ and scaffold N_50_ combination.

### Construction of genome-wide microsatellite database

Microsatellite motifs were identified using MISA [31] as described previously. The MISA output was processed using a custom PERL script modified from “P3in.pl” to prepare input for Primer3 [32]. Primer3 was installed on a local Linux server and run in batch mode with the following requirements: product size 100-280 bp; primer length 19-27 bp with optimal length 22 bp; primer annealing temperature Tm 56-61 °C with optimal Tm 58 °C; and primers must be at least 3 bp away from the microsatellite locus. The output was parsed with a custom PERL script modified from “p3out.pl” (http://pgrc.ipk-gatersleben.de/misa/primer3.html).

In-Silico PCR (http://genome.ucsc.edu/cgi-bin/hgPcr) was installed locally and run with maximum product size of 1kb to predict the amplified product(s) for each primer pair. The number of predicted amplicons for each primer pair was counted and those predicted to produce two or more amplicons were removed from the database. All text handling was performed using custom PERL scripts, and they are available upon request. 

### Experimental screening of microsatellite loci

 A subset of loci was tested experimentally. Four geographically representative *A. anomala* isolates, obtained from New Jersey, Ohio, Oregon, and Wisconsin, were used in the screening. DNA was obtained as described above. The M13(-21) mediated, nested PCR protocol [35] was used. Briefly, the M13(-21) sequence (TGTAAAACGACGGCCAGT) was fused to the 5’ end of each forward primer. The PCR was performed in 10 μl reactions containing 10 ng DNA, 160 nM each of FAM-labeled M13(-21) [FAM-M13(-21)] and reverse primer, 40 nM of forward primer, 0.2 mM dNTPs, 1x reaction buffer, and 0.2 U PerfectTaq DNA polymerase (5 PRIME). The forward and reverse primers first amplify their target. After the forward primer is exhausted, FAM-M13(-21) anneals to the M13(-21) sequence fused to the 5’ end of the forward primers. It then serves as the forward primer, and together with reverse primer, amplifies the product and provides fluorescence. As a first-pass screening, PCR was performed in 96-well plates without replicates. The PCR cycle parameters were as follows: an initial denaturation at 94 °C for 5 min was followed by 30 cycles of denaturation (94 °C, 30 s), annealing (56 °C, 45 s), and extension (72 °C, 45 s), followed by another 8 cycles of denaturation (94 °C, 30 s), annealing (53 °C, 45 s), and extension (72 °C, 45 s), and a final extension at 72 °C for 10 min. The PCR fragments were analyzed on ABI 3730 sequencer. Allele sizes were determined with the aid of software Peak Scanner version 1.0 (Life Technologies). 

### Population study and data analysis

 A collection of 30 isolates from the United States were screened using 11 polymorphic loci as described above. The data was analyzed using PowerMarker version 3.25 [36]. Summary statistics, such as major allele frequency, gene diversity [37], and polymorphism information content [38] were calculated for individual loci. Frequency-based genetic distance was calculated using shared alleles [39]. Isolates were clustered using the Unweighted Pair Group Method with Arithmetic Mean (UPGMA). The UPGMA tree was bootstrapped with 100 replicates. Consensus tree was computed using the program “consense” in software package PHYLIP version 3.695 (http://evolution.genetics.washington.edu/phylip.html). 

## Supporting Information

Table S1 **A genome-wide database of microsatellite loci in *A. anomala*.**
(XLSX)

Table S2 **Microsatellite motifs in *A. anomala* by motif type and number of repeats.**
(XLSX)

Table S3 **Results of experimentally screened microsatellite loci in *A. anomala* using four isolates from New Jersey, Ohio, Oregon, and Wisconsin.**
(XLSX)
